# Mental health promotion and prevention: Adjusting to the changing world: ***Selected Abstracts from the 99th EGPRN Meeting, 17–20 October 2024, Budapest – Hungary***

**DOI:** 10.1080/13814788.2025.2452583

**Published:** 2025-01-30

## Mental health promotion and prevention: Adjusting to the changing world

The Theme ‘Mental Health Promotion and Prevention: Adjusting to the Changing World’, particularly in the context of the COVID-19 pandemic, plays a pivotal role in addressing the profound impact of the pandemic on mental health globally. As the world navigates through unprecedented challenges brought about by the pandemic, it has become increasingly evident that mental health promotion and prevention efforts are more critical than ever.

In light of the COVID-19 pandemic and the ongoing conflicts, the conference emphasised the following key areas:

- Impact of the Pandemic on Mental Health;
- Innovative Strategies for Mental Health Support;
- Building Resilience and Coping Mechanisms;
- Addressing Mental Health Disparities;
- Supporting Frontline Workers and Healthcare Professionals;
- Policy Implications and Future Directions.

Overall, the conference served as a crucial platform for sharing knowledge, best practices, and lessons learned in promoting mental health and preventing mental illness in the context of a rapidly changing world and the ongoing challenges posed by the COVID-19 pandemic. By fostering collaboration and innovation, the conference aimed to contribute to the global effort to prioritise mental health and support the well-being of individuals and communities worldwide.

