# Supplementary material for: Mental health promotion and prevention: Adjusting to the changing world: Selected Abstracts from the 99th EGPRN Meeting, 17–20 October 2024, Budapest – Hungary
Source: Eur J Gen Pract. 2025 Jan 30;31(1):2452583. doi: 10.1080/13814788.2025.2452583 (PMC11784033; doi:10.1080/13814788.2025.2452583)
Supplement: Related texts

# Abstract: Mental health promotion begins at birth – Or earlier

**Authors:** Wilson Philip

**Affiliations:** Department of Family Medicine, University of Copenhagen

## Abstract

This talk provided a brief overview of the mechanisms by which *in-utero* factors and exposures in early life can influence brain development and thereby later mental and physical health. Epidemiological studies of mental health trajectories were discussed critically, along with their implications for Primary Care developmental screening, opportunistic child consultations, health promotion, and early intervention.

# Abstract: Low-intensity psychological interventions in primary care

**Authors:** György Purebl

**Affiliations:** Head of Institute of Behavioural Sciences, Faculty of Medicine, Semmelweis University Hungarian

## Abstract

In everyday medical practice, there are many situations that require some form of psychological support. Many medical complaints and physical illnesses are accompanied by psychological symptoms (most often anxiety, mood symptoms, irrational attitudes to therapy), which can have a decisive influence on treatment compliance and outcome and, if left untreated, can lead to mental disorders (e.g. depression, acute stress disorder, etc.). In addition, people with chronic illnesses very often have a comorbidity of some kind of mental disorder (most often depression). These situations make it necessary not only for psychiatrists and clinical psychologists, but also for all doctors (and health care workers in general) to have at their disposal psychological methods that can be applied quickly and easily in the time-limited everyday situations of medicine. Fortunately, these methods are now available and are collectively known as Low Intensity Psychological Interventions (LIPI). They have the following common characteristics: (1) easy and quick to learn (even from a manual), (2) do not require special psychological training, (3) time saving, and (4) easy to implement.

# Abstract: A novel approach in telemedicine – Bringing hybrid, contact- and telemedicine-based mobile healthcare services in rural Hungarian areas

**Authors:** Ábel Perjés, Rita Kovács, Márk Virág, Gergely Marovics, Luca Tóth, Barbara Sándor, Veronika Győri-Dani, Ferenc Nagy, János Sándor, Anita Pálinkás, Szilárd Redneki, Péter Maróti, Ferenc Vincze

**Affiliations:** Hungarian Charity Service of the Order of Malta, University of Debrecen Institute of Public Health and Epidemiology, University of Pecs Medical Skills Education and Innovation Center, Semmelweis University Department of Family Medicine, Budapest, Hungary

**Keywords:** Telemedicine, rural health, underserved population

## Abstract

**Background:**

Limited access to healthcare is one of the main problems effecting rural, socioeconomically underprivileged areas, such as the municipalities in the ‘Emerging settlements program’ in Hungary.

**Research questions:**

We aimed to prove the feasibility of telemedicine-assisted care for underprivileged populations.

**Method:**

The Hungarian Charity Service of the Order of Malta operates a mobile healthcare program (MHP) in 30 ‘Emerging Settlements’ in 5 clusters. MHP consists of 12 Mobile clinics: vans that are equipped with broadband internet connection, a high-resolution teleconference platform, devices enabling certain aspects of the physical examination via telemedicine, and additional diagnostic tools, such as point-of-care lab testing devices. A mobile clinic is operated by a trained nurse and a driver who meet the patients in person, while the medical doctor perform the consultation using the teleconference platform. The care focuses on screening for cardiovascular (CV) risk factors, manifest CV diseases and diabetes, and provide chronic care for those diagnosed. The mobile ultrasound clinic and the telespecialist network extend the delivered care beyond the realm of primary care.

**Results:**

We examined the care delivered at MHP in the period from 01.04.2023 to 31.10.2023. The 30 settlements had a total population of 15,294. 1,889 people (1429 adults and 460 children, 12.35% of the overall population) had at least one consultation in this period. The average number of care events was 2.44 for adults and 1.36 for children. Blood pressure and glucose levels of 1,430 patients were checked, resulting in 105 new hypertension and 26 new diabetes daignoses. 987 patients received a total of 2,177 referrals (1,024 within Maltese telemedicine providers and 1,153 to external healthcare providers). A significant, 10% decrease in relative referral frequency was detected (RR change: 0.9 [0.86; 0.95]).

**Conclusions:**

MHP can effectively deliver medical care, improve chronic disease management and enhance definitive treatment in the patients’vicinity in underprivileged regions.

# Abstract: Burnout syndrome and risk factors in trainees and fully qualified GPs: an observational study

**Authors:** Lorenzo Rizzotto, Lucia Luzi Crivellini, Andrea Cesaro, Giulio Rigon, Francesco Del Zotti, Sonia Zenari

**Affiliations:** Centro Studi Fimmg Verona, Monteforte d’Alpone 37032, Italy

## Abstract

**Background:**

Burnout represents a widespread social problem, particularly relevant in helping professions like GPs. Italy has the highest scores relating to burnout among European GPs. Understanding and improving the professional quality of life of GPs is crucial for the wellbeing of physicians and to ensure optimal quality of care.

**Research questions:**

The study aims to evaluate the working conditions of GPs in the Veneto region: what are the risk elements of burnout and the associated protective factors? Is there a difference between physicians in training and those already qualified?

**Method:**

An analytical-observational research was conducted through an online questionnaire for GPs of Veneto region and the organisation of five focus groups (three constituted of 8–12 fully qualified GPs and two of 15–20 training GPs). The questionnaire collected demographic data, information on working habits, and professional wellbeing using the Professional Quality of Life Scale (ProQOL-Health). The focus groups were used to deepen the results and gather qualitative data on the personal experiences of GPs regarding burnout and coping strategies.

**Results:**

499 questionnaires were completed, 241 by training GPs and 260 by trained GPs. High levels of burnout were reported both in training GPs (45%) and in fully qualified ones (35%), in spite of the lower number of assisted patients by the trainees. The Perceived Support by the trainees and other GPs was, respectively, of 38% and 30%, and the Compassion Satisfaction (CS) scores were 60% and 55% in the two groups. GPs with nursery and secretary desk resulted in higher risk of developing burnout syndrome, while there was a positive correlation between burnout syndrome and CS.

**Conclusions:**

Burnout rates were comparable between training and trained GPs. Excess of working hours and higher number of employees was the greatest risk factors. From the focus groups, prolonged multi-channel contactability and bureaucracy were great stressors. Among training GPs, Perceived Support resulted a protective factor.

# Abstract: Strategies of German general practitioners in dealing with depressive disorders in primary care patients – A mixed-methods study

**Authors:** Ildikó Gágyor, Sandra Parisi, Laura Hofner

**Affiliations:** Department of General Practice, University Hospital Würzburg, Wuerzburg 97080, Germany

**Keywords:** Depressive disorders, general practice, mixed-methods study design, survey, in-depth interviews

## Abstract

**Background:**

Depression is a global health issue that challenges general practitioners (GPs) when patients seek medical care. In Germany, psychiatrists and psychotherapists are involved in ambulatory care. In this context, the role of GPs in treating depression is not yet fully understood.

**Research questions:**

What strategies do GPs use to manage patients with depression? How do GPs perceive their role?

**Method:**

In January 2021, we sent a survey to 1800 randomly selected Bavarian GPs including an invitation to an in-depth interview (mixed-methods design). The 53 items comprised patient management (e.g. consultations, diagnostics, medication) and demographics. Survey data was analysed descriptively. Interviews were analysed using Kuckartz’s content analysis. We employed a triangulation protocol to integrate quantitative and qualitative data.

**Results:**

Overall, 471 GPs (45.4% female) completed the survey, and 20 participated in an interview. GPs reported referring 6 out of 10 patients to a psychotherapist (mean = 6.0, SD = 2.4), referring 6 out of 10 to a psychiatrist or neurologist (mean = 5.6 SD = 2.5), prescribing antidepressants to 5 out of 10 (mean = 4.7, SD = 2.6) and prescribing herbal drugs to 3 out of 10 (mean = 2.8, SD = 2.4). Interviews revealed that patients’ expectations and concerns guide GPs’ decisions. GPs provide psychosocial support in cases of social burden due to family conflicts and work stress. GPs see themselves as gatekeepers, patients’ advocates, comforters, and problem-solvers, but also describe themselves as sole primary care providers, challenged by limited time and referral options. During the COVID-19 pandemic, GPs noticed rising mental health issues and a decline in supporting healthcare structures. At the same time, GPs had more time for mental health care as they had fewer routine medical tasks to perform.

**Conclusions:**

Psychosocial guidance is a key strategy for treating depression in primary care. GPs are committed to providing a patient-centered therapeutic approach, yet often feel constrained by the existing healthcare structures.

# Abstract: Loneliness, problematic smartphone use and screen time among medical students in Germany

**Authors:** Lukas Liebig, Hendrik Berth, Erika Balogh, Béla Birkas, Nora Faubl, Erika Zelko, Henna Riemenschneider

**Affiliations:** Department of General Practice, Faculty of Medicine and University Hospital Carl Gustav Carus, Technische Universität Dresden, Dresden, Germany

**Keywords:** Loneliness, proplematic smartphone use, post pandemic, medical education, mental health, digitalisation

## Abstract

**Background:**

In the course of the COVID-19 pandemic, loneliness, smartphone addiction and screen time have increased, especially among young people. The extent to which medical students in Germany are currently affected by this as well as the relationship between loneliness, problematic smartphone use (PSU) and screen time have not yet been examined.

**Research questions:**

(1) What is the prevalence of loneliness, problematic smartphone use and screen time among medical students in Germany after the COVID-19 pandemic? (2) How can the relationship between loneliness, problematic smartphone use and screen time be described?

**Method:**

Medical students at the Technical University of Dresden took part in a voluntary, pseudonymised online cross-sectional survey in 2022. Group comparisons, correlations, mixed-model analyses and mediation analyses were calculated to describe the relationship between loneliness (3-Item UCLA loneliness scale), PSU (SAS-SV) and screen time (h per day).

**Results:**

580 medical students (*M* = 24 years, 73% female) were included in the analysis. The students had an average loneliness score of *M* = 4.83 (SD =2.8) and 17.1% were identified as lonely. The average SAS-SV score was *M* = 24.33 (SD = 8.3), with 17.2% identified as smartphone-dependent. The students had an average screen time of *M* = 7.65 h (SD =2.4) per day. A higher level of loneliness was associated with increased PSU (*r* = 0.211, *p* < 0.001) and increased screen time (*r* = 0.108, *p* = 0.009). The relationship between loneliness and increased screen time during leisure was partially mediated by PSU.

**Conclusions:**

Loneliness, problematic smartphone use and increased screen time are widespread and significantly associated among medical students in Germany. The results are relevant for future interventions to promote psychological and physiological well being of medical students.

# Abstract: Benzodiazepine use and incident cancer – A population-based Cohort study

**Authors:** Yochai Schonmann

**Affiliations:** Clalit Health Services, Tel Aviv, Israel

**Keywords:** Benzodiazepines, malignancy, cancer, population-based, routinely collected data

## Abstract

**Background:**

Both long-term and sporadic use of benzodiazepines and Z-drugs are associated with various adverse effects, yet they are frequently prescribed for insomnia and anxiety in primary care. Although a link between their use and cancer incidence has been suggested, long-term population-based data is needed.

**Research questions:**

Does the use of benzodiazepines and related drugs predict future incident cancer?

**Method:**

A population-based historic cohort study was conducted, following all Clalit members aged 40–70 from 2006 to 2022. Cox regression models were fitted to assess the association between sporadic and chronic benzodiazepine use at baseline (based on cumulative claims during 2005) and subsequent incident cancer. Results were reported both in crude form and adjusted for age, sociodemographic characteristics (sex, ethnicity, socioeconomic status, peripherality), and baseline smoking and obesity.

**Results:**

We included 1,027,582 Clalit members without any malignancy diagnosis at baseline. The mean age was 53.0 years (±SD =8.1), and 52% were female. Of the participants, 102,619 (10.0%) were sporadic users of benzodiazepines at baseline, and 29,507 (2.9%) were chronic users, with at least nine monthly claims of a benzodiazepine in 2005. Over a median follow-up of 17.0 years, there were 153,956 cases of new cancer diagnoses. Sporadic use of a benzodiazepine was associated with a slightly increased incidence of cancer (adjHR 1.07, 95% CI 1.05–1.08, *p* < 0.0001), while chronic use was associated with a higher risk (adjHR 1.13, 95% CI 1.10–1.16, *p* < 0.0001), compatible with a linear association (*p* < 0.0001). The association remained robust regardless of age but was stronger among younger cohort participants. Subsequent analysis confirmed the same association for both anxiolytic and hypnotic medications but not for the use of Z-drugs (adjHR 1.03, 95% CI 0.99–1.07, *p* < 0.0001 for sporadic use, and adjHR 0.99, 95% CI 0.90–1.08, *p* < 0.0001 for chronic use).

**Conclusions:**

The use of benzodiazepines, but not Z-drugs, was associated with incident cancer, with evidence supporting a dose-response relationship.

# Abstract: Internal medicine physicians’ struggle with family planning and infertility

**Authors:** Isa Egger, Christa Nater, Jarmila Zdanowicz, Karolina Kublickiene, Sven Streit, Jeanne Moor

**Affiliations:** Institute of Primary Health Care (BIHAM), University of Bern, Bern, Switzerland

**Keywords:** Internal medicine physicians, family planning, infertility

## Abstract

**Background:**

Physicians in Europe are more likely to experience family planning conflicts than other university graduates. Furthermore, literature from the US suggests that female physicians have higher infertility rates than the general population. However, literature on these topics for Switzerland, a country with a well-funded healthcare system, is lacking.

**Research questions:**

We aimed to determine the prevalence of infertility and age at birth of first child among Swiss female Internal Medicine (IM) physicians. Furthermore, we determined the intent to delay having children among medical students and IM physicians.

**Method:**

We used a cross-sectional web-based anonymous survey, which was distributed by e-mail among Swiss IM physicians via IM societies and hospital departments. Medical students at the University of Bern were contacted by the medical faculty. Data about the following questions were collected and analysed by descriptive statistics: Demographics, self-reported infertility, age at birth of first child, and delaying having children.

**Results:**

This study included 682 physicians (59% women) and 321 medicals students (70% women). Median age of physicians was 32 years (interquartile range (IQR) 30–42) and of medical students 24 years (IQR 23–25). Among 148 female physicians who had tried to conceive, 41 (28%) fulfilled the criteria of infertility. Median age at birth of first child was 31 years (IQR: 29–35). Among those physicians who already have children, 42% of female and 22% of male physicians delayed having children (*p* ≤ 0.001). Among medical students, 63% of female and 44% of male students are planning to delay having children (*p* = 0.013).

**Conclusions:**

Swiss physicians’ estimated infertility prevalence is roughly twice as high as the general population’s (28% vs. 10–15%), possibly due to delaying having children. Our results suggest that infertility awareness, more flexibility for family planning and workplace restructuring in medicine in Switzerland is required to accommodate physicians’ family planning needs.

# Abstract: Medical decision-making challenges among ethnic minority populations in Belgium: A focus group study

**Authors:** Flore Vermijs, Amina Yakhlaf, Josefien Van Olmen, Paul Van Royen, Katrien Bombeke, Sarah Van De Velde, Edwin Wouters, Veerle Buffel, Nina Van Eekert

**Affiliations:** University of Antwerp, Antwerpen, Belgium

**Keywords:** Medical decision-making, culturally sensitive care, focus group discussions

## Abstract

**Background:**

Cultural differences can influence the process of medical decision-making (MDM) between general practitioners (GPs) and patients with an ethnic minority background, potentially threatening the quality of person-centred care.

**Research questions:**

What are the challenges related to MDM among patients with an ethnic minority background, from the perspectives of patients and GPs in Belgium?

**Method:**

Three focus group discussions with patients (or their representatives) with a migration background from Turkey (*n* = 6), Morocco (*n* = 6) and sub-Saharan Africa (*n* = 7), and two with GPs (*n* = 13) were conducted in Belgium between November and December 2023. Reflexive thematic analysis of the transcripts integrated medical, sociological and deontological/legal perspectives.

**Results:**

Challenges arise from differences in conceptions about diseases and their corresponding treatment, discordant expectations regarding patient involvement in their own health and MDM, and providers’ lack of knowledge and understanding of patients’ preferences regarding disclosure of medical information and treatment. Moreover, close family involvement can affect the MDM process at multiple stages. Challenges were most prevalent in MDM about prevention, non-communicable diseases, mental health, sexual and reproductive health, and end-of-life care. Importantly, the intersection with other factors, such as language proficiency, education, socio-economic status, length of residence in Belgium, gender dynamics, religious affiliations, as well as Belgian legislation related to MDM should be recognised.

**Conclusions:**

The analysed patient and GP perspectives enrich our understanding of the complex cultural dynamics surrounding MDM. This study highlights the importance of working towards culturally sensitive healthcare practices that accommodate the diverse needs and preferences of ethnic minority populations in the MDM process, while taking the deontological/legal framework as well as the educational and supportive needs of their GPs into account.

# Abstract: Association between affective temperaments and the severity and the extent of coronary artery disease as obtained by coronary CT angiography

**Authors:** Barbara Sipos, Milán Vecsey-Nagy, Borbála Vattay, Melinda Boussoussou, Zsófia Jokkel, Sarolta Borzsák, Ádám Jermendy, Alexis Panajotu, Xenia Gonda, Zoltán Rihmer, Béla Merkely, Bálint Szilveszter, János Nemcsik

**Affiliations:** Semmelweis University, Budapest, Hungary

**Keywords:** Affective temperaments, coronary artery disease, coronary CT angiography

## Abstract

**Background:**

Affective temperaments are documented predictors of psychopathology, but cumulative data also suggest their association with coronary artery disease (CAD).

**Research questions:**

We aimed to evaluate role of affective temperaments in relation to surrogate semiquantitative markers of coronary plaque burden, as assessed by coronary CT angiography (CCTA). We also compared the application of the 110-item Temperament Evaluation of Memphis, Pisa, Paris and San Diego Autoquestionnaire (TEMPS-A) and of the 40-item TEMPS-A autoquestionnaire.

**Method:**

We included 351 patients who were referred for CCTA due to suspected CAD. All patients completed the TEMPS-A. The severity and extent of CAD was evaluated by CCTA, applying semiquantitative plaque burden scores, notably Segment Involvement Score (SIS) and Segment Stenosis Score (SSS). Logistic regression analyses were performed to define the predictors of CAD severity and extent.

**Results:**

Regarding the scores evaluated by TEMPS-A consising of 110 questions, a significant inverse association was found between hyperthymic temperament score and SSS (*β* = –0.143, (95%CI: –0.091 to −0.004), *p* = 0.034), in men. Compared to the TEMPS-A 110-item version, applying the abbreviated version – containing 40 questions – showed a significant relationship between affective temperaments and SSS or SIS was found in case of both sexes. Concerning men, hyperthymic temperament was demonstrated to be independent predictor of both SSS (*β* = –0.193, (95%CI: –0.224 to −0.048), *p* = 0.004) and SIS (*β* = –0.194, (95%CI: –0.202 to −0.038), *p* = 0.004). Additionally, we proved, that significant positive association between irritable temperament and SSS (*β* = 0.152, (95%CI: 0.002–0.269), *p* = 0.047) and SIS (*β* = 0.155, (95%CI: 0.004–0.221), *p* = 0.042) also exists among women.

**Conclusions:**

Assessment of affective temperaments could offer added value in stratifying cardiovascular risk for patients, beyond traditional risk factors in general practice.

# Abstract: A comparative randomised trial among different types of low and high power laser therapy, associated with steroid or visco-elastic joint injection under ultrasound guidance in the management of knee osteoarthritis in primary care

**Authors:** Mihai Iacob

**Affiliations:** a The European Ultrasound Workıng Group/Euvekus/Eadus; b Tımıs Socıety of Famıly Physıcıans; c Timis Medical Chamber; d The Romanian National Society of Family Medicine (SNMF), Tımısoara 300150, Romania

**Keywords:** Knee ultrasonography, knee osteoarthritis, Low-Power-Lasertherapy (LLLT), High-Intensity-Lasertherapy (HILT), Steroid Joint Injection (SJI), Hialuronic acid joint injection (HAI), knee pain management, knee POCUS applications

## Abstract

**Background:**

Knee osteoarthritis is a degenerative arthropathy, progressive, disabling and affecting older individuals. It is characterised by joint stiffness and pain and structural changes with degradation of joint cartilage, bone remodelling, and inflammatory changes. This study aims to present the results of Low Power Laser Therapy (LLLT), combined with local steroid infiltration, compared treatment with High-Intensity Laser Therapy (HILT) in infra-red emission, followed by hyaluronic acid injection with ultrasound guidance.

**Research questions:**

Which type of therapy is more effective in knee osteoarthritis management?

**Method:**

Inclusion criteria were patients with moderate or severe knee osteoarthritis in different stages. We conducted a prospective unblinded trial (RCT) over 3 years, on 750 patients, using two laser devices (semiconductor source). The steroid used in Joint Injections (SJI) was dexamethasone. We used knee-ultrasonography for diagnostic and therapeutic purposes in all cases. The cases studied were divided into three groups: the Control Group included patients without medication; the second group included patients treated with LLLT with SJI; and the third group included patients treated with HILT followed by joint-injection of the hyaluronic acid (HAI). Elements evaluated by each patient were: pain on a visual analog scale (VAS), a motion-functional-scale (MFS) of the knee disability, and the WOMAC-Score.

**Results:**

The healing rate for the first, second and third groups was, respectively, 10%, 77% and 88%. Analysis of risk and data obtained on patients by VAS/WOMAC/MFS-scales, before and after treatment, within each group was compared by Student’s *t*-test, and among all three groups after the final evaluation of patients by ANOVA, *p* < 0.001. The results of 2x2 Contingency-Tables were: Relative-Risk (RR):0.46 (Benefit), Odds Ratio (OR):0.30-between LLLT/SJI versus Control Group and RR between HILT/HAI was 0.20, 95%CI = 0.13–0.29, Odds Ratio = 0.11, *p* < 0.0001.

**Conclusions:**

The combination of HILT and HAI significantly improved the outcome by 48% compared to conventional therapy and may be considered the most effective treatment. HILT followed by a joint viscoelastic injection was more effective than LLLT with SJI in knee osteoarthritis management.

# Abstract: “VoluntariaMente” – The Impact of the individual social role on the improvement of mental health

**Authors:** Patrícia Tuna, Sofia Almeida, Ana Gonçalves, Ana Sara Ferreira, Francisco Coelho, Pedro Couto, Bárbara Reis

**Affiliations:** Health Center of Valongo, Porto, Portugal, Resende, Portugal

**Keywords:** Mental health, anxiety, depression, volunteering, primary health care

## Abstract

**Background:**

Mental health problems are very common and represent a significant proportion of health-seeking contacts in primary health care. Given the prevalence and impact of mental health disorders, it’s important to improve methods that promote social integration and well-being of these patients. Studies have shown that volunteer work promotes not only mental health, but also social inclusion, and is related with lower anxiety and depression symptoms. Taking into account the PERMA model (Seligman, M.) who proposes five pillars on which psychological well-being and happiness are based – Positive Emotion, Engagement, Relationships, Meaning and Accomplishments – a methodology was created that aims to foster experiences that stimulate the psychological well-being and happiness of participants.

**Research questions:**

This study aims to evaluate the effects of volunteer work handled by people suffering with anxiety and/or depression disorders on their disease’s scores.

**Method:**

Patients from a Portuguese Family Healthcare Unit medicated with anxiolytics and/or antidepressants, in order to treat their anxiety and/or depression disorders, were selected and integrated in a volunteering project that lasted a year. Anxiety symptoms were measured by the Hamilton Anxiety Scale, and depression scores were quantified by the Patient Health Questionnaire-9, both before and after the intervention.

**Results:**

Thirty-three participants were selected and 25 were included in the study. The majority were female, over 50 years old, and performed an average of 48 h of volunteering. There was a statistically significant reduction in levels of anxiety or/and depression scores after the intervention, regardless of the number of volunteer hours performed.

**Conclusions:**

Qualified and accompanied volunteer work had a positive effect on the participants, significantly lowering anxiety and depression scores.

# Abstract: ‘I am confident … until patient X presents’ – A qualitative meta-synthesis to understand primary healthcare professionals experience with urinary tract infections

**Authors:** Henrike Kleuser, Francine Toye, Luca Ghirotto, Sandra Parisi, Alice Serafini, Maria Chiara Bassi, Felix Kannapin, Johannes Liese, Ildikó Gagyór, Peter Kurotschka

**Affiliations:** Department of General Practice, University Hospital Würzburg, Würzburg, Germany

**Keywords:** Management of UTIs, qualitative research, meta-synthesis

## Abstract

**Background:**

Urinary tract infections (UTIs) are among the most common infections encountered in primary care. Qualitative research shows that numerous factors such as prior experiences, habits, patient expectations, time constraints, or available resources may influence how Primary Care Professionals (HCPs: physicians, nurses, and other healthcare professionals) manage UTIs. Gaining insight into their perspectives facilitates better healthcare provision.

**Research questions:**

What are the experiences of HCPs when managing patients with UTIs? What aspects do HCPs consider when treating adults and children with UTIs?

**Method:**

We systematically searched PubMed, Embase, PsycINFO, and CINAHL from inception to October 7th, 2022, to retrieve qualitative research focusing on HCPs’ experiences in UTI management. We synthesised findings of included studies using Braun and Clarkés reflexive thematic analysis, and subsequently applied GRADE-CERQual to rate confidence in our findings. PROSPERO registration: CRD42022301250.

**Results:**

We retrieved 2,794 records, reviewed 140 studies in full, and selected 20 studies from nine different countries reporting the experiences of 389 HCPs. The theme ‘I am confident …until patient X presents’ illustrates that HCPs approached UTI consultations with a high level of confidence due to their familiarity and experience. However, confidence waned when particular groups of patients (children, recurrent UTIs, elderly patients, and male patients) presented. HCPs then reported uncertainty for initial diagnosis and treatment, fearing deterioration of symptoms and treatment failure. The theme ‘I have to balance patient preferences and antimicrobial resistance’ describes that HCPs carefully weighed individual risk factors against the danger of increasing resistance, acting in the patients’ and communities’ best interest. ‘I am a victim of an overstretched healthcare system’ describes how time-constraints, shortage of staff, limited resources, and reimbursement of consultations influenced the management of UTIs.

**Conclusions:**

HCPs are confident in managing UTIs until patients with non-specific symptoms present. Patient preferences, antimicrobial stewardship efforts and available resources influence the management of UTIs.

# Abstract: Risk factors for poor prognosis in outpatients with urinary tract infection: A systematic review and meta-analysis

**Authors:** Peter Kurotschka, Felix Kannapin, Andreas Klug, Maria Chiara Bassi, Ildikó Gágyor, Mark H. Ebell

**Affiliations:** Department of General Practice, University Hospital Wurzburg, Wurzburg, Germany

**Keywords:** Urinary tract ınfections, prognostic factors, outpatients, primary care, systematic review, meta-analysis

## Abstract

**Background:**

Observational studies identified several risk factors of poor prognosis in outpatients with urinary tract infection (UTI). These findings have never been systematically reviewed.

**Research questions:**

Which are the risk factors for poor prognosis in adult outpatients with UTI?

**Method:**

We searched Medline, Embase, Scopus, Cinahl, and Web of Science through November 2023. In parallel, two reviewers screened all records, abstracted study characteristics, risk factor and outcome data, and assessed risk of bias. We grouped outcomes into two categories of increasing severity: re-consultation and hospital admission. For risk factors reported by three or more studies, we performed random–effects meta-analysis of relative risks (univariate data) and adjusted odds ratios (aORs, multivariate data). We assessed heterogeneity visually and using the I2 statistic.

**Results:**

We included 36 studies with 1,948,814 adults aged 32 to 77 diagnosed with cystitis or pyelonephritis (PN). The only independent predictor of the outcome reconsultation was older age: the likelihood of experiencing this outcome for every additional 10 years of age increases by 18% (aOR 1.18). Independent predictors for hospital admission include age >64 (aOR 3.51), male sex (aOR 1.41), and diabetes (aOR 1.34) for both patients with cystitis and with PN. Increased procalcitonin (PCT, aOR 5.12), low systolic blood pressure (aOR 3.29), fever >38 °C (aOR 2.08), C-reactive protein (aOR 1.62), and serum creatinine >1.1 mg/dl (aOR 1.56) were also significantly associated with hospital admission in patients with PN.

**Conclusions:**

This is the first meta-analysis assessing risk factors for adverse outcomes in outpatients with UTI, either with acute cystitis or PN. We focused on clinical features, patient characteristics, and tests that are often available in the primary care setting. Until more evidence on prognosis of patients with UTI is available, clinical guidelines should take these risk factors into account when defining populations at risk of deterioration.

# Abstract: The health care worker well-being survey (WBS): An assessment of the impact of well-being and climate change on health care quality improvement, Tajikistan, 2023

**Authors:** Surayo Pulatova, Tahmina Jaborova, Halima Boboyeva, Gulnora Razykova

**Affiliations:** Tajik Medical Institute/Abt Global, Dushanbe, Tajikistan

**Keywords:** Health care workers, well-being, professional burnout, emotional state, stress level, climate change, quality improvement, Tajikistan

## Abstract

**Background:**

A comprehensive health care worker (HCW) well-being survey was conducted in Tajikistan by Healthy Mother, Healthy Baby (HMHB) Activity. The article highlights the significance of prioritising the well-being and mental health of HCW, particularly in the context of providing quality care for women and children. The research demonstrates how professional burnout and stress as well as climate change can diminish quality of healthcare. Lessons learned validate how enhancing the well-being and support of HCWs contributes to improving quality of care, preventing burnout and improving job retention.

**Research questions:**

Does work environment and climate change impact Tajik HCW well-being and negatively impact quality of care?

**Method:**

HMHB received legal consent from the U.S. Health Resources and Services Administration and the Bureau of Primary Health Care to use and digitalise the survey tool in Tajik and Russian. An additional section on impacts of climate change was added. The survey was conducted in 12 HMHB-supported districts of Bokhtar Zone of the Khatlon region, Tajikistan. 252 respondent were surveyed.

**Results:**

7.1% of HCWs indicated that they feel professional burnout. Of these, respondents answered that they feel tired even before coming to work (49.4%), need more time to rest than before (52.6%), often feel emotionally exhausted while working (45.8%) and usually feel overwhelmed and tired after work (76.1%). The high frequency of communication with patients was noted by 74.1% of HCWs, and is one of the reasons leading to psychological stress and professional burnout. 72.8% of respondents confirmed that climate change significantly affects their mental and physical health.

**Conclusions:**

The survey provided multilevel data to help identify health system strengths and weaknesses affecting HCW burnout and exhaustion which can lead to increased medical errors. The findings from the survey offer valuable data that can be used to shape policies and approaches to improve healthcare quality and outcomes.

# Abstract: Patterns and associated factors in health service utilisation among high school students in Turkey

**Authors:** Cigdem Apaydin Kaya, Beyza İrem Birinci, Emine Erçil Malcık, Ahmet Hamdi Akgündüz, Ayceren Taşan, İrem Gelgeç, Nuran Özkan

**Affiliations:** Family Medicine, Marmara University, Istanbul, Türkiye

**Keywords:** Adolescents, primary health care centres, health care utilisation, mental health

## Abstract

**Background:**

It is crucial for adolescents to benefit from health services, especially primary health care services to promote health and prevent diseases.

**Research questions:**

What is the health care utilisation patterns of high school-aged adolescents?

**Method:**

This descriptive study took place in Istanbul, February 2024. The research population consisted of students attending a high school. Efforts were made to include the entire population in the study. A survey was conducted to the students via Google Forms, including their sociodemographic characteristics, diseases, medications, nutrition and exercise habits, risky behaviours, two-question depression screening questions, and the frequency and reasons for using health services. Data were analysed using chi-square and One-Way ANOVA, *p* < 0.05 was considered statistically significant.

**Results:**

675 people (mean age of 15.84 ± 1.33) participated in the study. 13.03% of them had a chronic disease and 27.70% were on regular medication. In the last year 96.44% of the participants had sought services from a health institution (private hospital 40.89%; public hospital 40.74%, family health centres 38.67%, emergency services 19.11%, training and research hospital 17.63%). Those with low income were more likely to visit public hospitals than those without (*p* = 0.041). The most common reasons for applying to a health institution are cough (43.85%), sore throat (40.44%) and runny nose (39.70%). The participants first consult their families about their health problems (65.92%), then internet (13.63%) and a physician (13.03%). The individuals at risk of depression reported that they use the internet more frequently for health problems and more likely to share their thoughts with their families compared to those not at risk (14.4%, 6.7%; *p* = 0.016; 60.9%, 50.8%; *p* = 0.042).

**Conclusions:**

Our research indicates that high school students primarily utilise health services for acute issues and tend to use hospitals instead of primary health care centres for health issues. However, there is a significant demand for counselling on preventive services, particularly for mental health concerns.

# Abstract: Hospital-at-home costs and outcomes: A cohort study

**Authors:** Ilan Yehoshua, Sharon Baruch Gez, Bar Cohen, Beatrice Hemo, Angela Irony, Khaled Abou Houssien, Omri Shental, Limor Adler

**Affiliations:** Israel Association of Family Physicians, Sde Yoav, Israel

**Keywords:** Home hospitalisations, cellulitis, urinary tract infection, pneumonia

## Abstract

**Background:**

The challenges posed by increased life expectancy, ageing populations, and chronic morbidity have become pressing concerns for healthcare systems globally. Simultaneously, there is a documented reduction in the number of hospital beds, facilities, and resources. Hospital care and services constitute the single most significant expenditure in healthcare and are significantly higher than community healthcare services, where comparable.

**Research questions:**

This study aims to evaluate and compare health outcomes and costs between home hospitalisation and traditional hospitalisation for three common diagnoses – cellulitis, urinary tract infection (UTI), and pneumonia.

**Method:**

In this retrospective cohort study, we followed 1,311 patients in home hospitalisation and 992 patients in traditional hospitalisation. Patients were hospitalised between 1 January 2019 and 31 December 2020 for cellulitis, UTI, and pneumonia treatment. We evaluated healthcare costs, length of hospitalisation, referrals for further medical services, and mortality.

**Results:**

In this study, we were able to establish that home hospitalisation is non-inferior to traditional hospitalisation in metrics of mortality, rehospitalisations, and emergency department (ED) referrals for three common diagnoses – cellulitis, pneumonia, and UTI. In terms of hospitalisation length, home hospitalisations were shorter for pneumonia and cellulitis compared to traditional hospitalisation (5.01 vs. 6.05 days, *p*-value 0.001 and 5.3 vs. 6.1 days, *p*-value <0.001, respectively). Likewise, for cellulitis and pneumonia, home-hospitalised patients had fewer ED referrals compared to traditional hospitalisation (13.5% vs. 19.8%, *p*-value 0.002 and 13.7% vs. 24% *p*-value <0.001, respectively), and lower associated costs (7,366 vs. 11,401 New Israeli Shekel (NIS), *p*-value 0.001, and 6,869 vs. 11,326 NIS, *p*-value 0.001).

**Conclusions:**

Home hospital models may be a viable and even superior alternative for suitable patients for three common infectious diseases – cellulitis, pneumonia, and UTI – given an appropriate infrastructure.
